# The possible importance of income and education as covariates in cohort studies that investigate the relationship between diet and disease

**DOI:** 10.12688/f1000research.6929.2

**Published:** 2016-05-18

**Authors:** Norman Temple

**Affiliations:** 1 Centre for Science, Athabasca University, Alberta, Canada

**Keywords:** Cohort studies, socioeconomic status, covariates

## Abstract

Background:  Many cohort studies have been carried out that have provided information on the relationship between diet and health-related outcomes. Omission of important covariates during multivariate analysis may give rise to error due to residual confounding. A possibly important covariate is socioeconomic status (SES) as this is related to both diet and health.

Objective: To determine the frequency with which different measures of SES are included as covariates during multivariate analysis of cohort studies that investigated the relationship between diet and health.

Methodology:  An analysis was carried out of 76 randomly selected papers from 66 cohort studies. The papers covered many dietary variables and a wide variety of diseases/health-related outcomes. The cohort studies were carried out in many different locations and the subjects varied widely in age.

Results:  Approximately two-thirds of the papers (65.8%) used at least one measure of SES as a covariate. Education was used most often (60.5% of papers), followed by income (14.4%) and social class (2.6%). More than one measure of SES was used in 11.8% of papers.

Conclusions:  Failure to include income (or another measure of present SES, such as occupation) may be a common source of error in cohort studies. Over-reliance on education may be particularly important as it is likely to be a weaker measure of *present* SES than is income. There is a need for more research on this question. SES in childhood is almost never included in multivariate analysis in cohort studies carried out on adults. This could also play a significant role in disease risk in middle age or later. Very little is known regarding whether this is also a source of residual confounding.

## Introduction

Socioeconomic status (SES) is related to diet and health. It is well established that affluent people have superior health compared to poorer people ^1^. A major part of the explanation for this is that people with high SES generally follow a healthier lifestyle ^1^. Of particular note, many studies have reported that people with a higher SES consume a more nutritious diet ^2^. This pattern has been consistently reported in the USA, Canada, and many European countries, and is seen with different indicators of SES – education, income, and occupation.

This suggests that income, or other indicators of SES, may be a relevant factor in cohort studies. It follows, therefore, that failure to include SES in multivariate analysis of findings from cohort studies may be a source of error due to residual confounding. The potential importance of this was shown by an analysis of data from the British Women’s Heart and Health Study. Adjusting for a range of factors that indicate SES, both those in childhood and adulthood, attenuated the relationship between the plasma concentration of vitamins C and E and risk of coronary heart disease (CHD) in adults aged over 60 years ^3, 4^.

The analysis described here was carried out in order to investigate the extent of this potential source of error.

## Methods

An analysis was carried out of papers that reported the findings of cohort studies. The main inclusion criteria were, first, the papers were published in journals in the year 2000 or later, and, second, they reported findings on the relationship between dietary intake and health-related outcomes, such as body weight or disease. The papers were found using two main search strategies: (1) they were cited in various meta-analyses; and (2) by searching journals that cover the areas of nutrition, health, and medicine but with the search restricted to issues published in late 2013. A list of all papers used in this study is included in the Supplementary material.

Each paper was studied and key information was extracted. In particular, a record was made indicating whether the risk ratios were adjusted for factors that indicate SES, such as education and income. Where available, information was also extracted that reported associations between SES and dietary variables (e.g., whether study subjects with more education consumed more fish). In addition, for comparative purposes a record was kept of whether alcohol, physical activity, and hypertension were included as covariates.

Many cases were found where two or more papers were based on the same cohort study. In those cases each paper was evaluated. However, only one of the papers was included here unless, with respect to the covariates that are the focus of this paper, the papers used different covariates in the multivariate analysis. Papers were also included if they contained relevant information on the association between SES and dietary variables. After these exclusions and inclusions 76 papers from 66 cohort studies were included in the final analysis. Ten were published in the years 2000 to 2003, 15 during 2004 to 2007, 27 during 2008 to 2012, and 24 during 2013. The study is therefore based on a convenience sample of cohort studies rather than a systematic review. Nevertheless, because of the large number of cohort studies included, the findings are likely to be representative of cohort studies published in recent years.

## Results

The findings reported here are based on 66 cohort studies of which 52 were carried out on adults and 14 on children (age <18 years). They were carried out in the USA (30), Europe (28), Asia (5), and Canada (3).

The analysis included 76 papers. They covered a wide variety of diseases/health-related outcomes, including body weight or another measure of adiposity (25), cancer (21), type 2 diabetes (5), and all-cause mortality (16). Cardiovascular disease was studied in 33 papers, of which 19 looked at all forms of CVD combined, 13 at CHD, one at both CVD and CHD, and two at stroke. These 76 papers covered many aspects of the diet, including sugar-sweetened beverages (18), diet patterns (14), multivitamin supplements (8), fish (7), milk (7), sodium (6), and meat (4). The great majority of the papers covered only one dietary variable; the main exception was studies on beverages where different beverages were often included in the same paper. Physical activity was included as a covariate in 56 of the 76 papers (73.7%), alcohol in 43 of the 61 papers on adults (70.5%), and hypertension or blood pressure in 21 of the 33 papers on CVD (63.6%). At least one measure of SES was included as a covariate in 50 of the 76 papers (65.8%). Education was by far the most common (60.5%), followed by income (14.4%) and social class (2.6%). Several papers (11.8%) included more than one measure of SES.

Papers based on studies of children used a measure of SES with a similar frequency to those on adults. Parental SES was used in those studies.

Many of the papers reviewed here reported on the association between SES and diet. In general, people with more education consumed a diet that is associated with better health. This includes more fish ^5– 7^, less meat ^8, 9^, as well as a healthier overall dietary pattern ^10– 13^. In addition, some papers reported that those with more education consume more sugar-sweetened beverages ^14, 15^, more fruit juice ^14, 16^, and more sodium ^17, 18^. A particularly clear trend is the association between education and consumption of multivitamin supplements ^19– 24^.

A small number of the papers reported on the association between income and diet. Consistent with the above findings higher income is associated with eating more fish ^6, 7^ and a healthier overall dietary pattern ^12^.

## Discussion

The methodology used here has strengths and weaknesses. The papers analyzed were mostly published in the last few years (51 of the 77 papers appeared between 2008 and 2014 while the remainder appeared between 2000 and 2008). They cover a wide range of dietary components and outcomes. Sixty-four of the 66 cohort studies were carried out in the USA, Canada, Europe, or Japan. The findings are therefore likely to be representative of cohort studies published in recent years that investigated the relationship between diet and risk of a wide variety of diseases and health-related outcomes. However, as the papers analyzed were randomly selected, there may be a degree of selection bias.

Approximately two-thirds of the papers (65.8%) used at least one measure of SES as a covariate. The most commonly used one was education (used in 60.5% of papers), followed by income (14.4%) and social class (2.6%). Some papers (11.8%) used more than one measure of SES. The frequency of inclusion of SES as a covariate is similar to that seen for the other covariates that were looked at here (73.7% for physical activity, 70.5% for alcohol in papers on adults, and 63.6% for hypertension or blood pressure in papers on CVD).

Many of the papers provided information on the relationship between SES and diet. The findings reveal that people with more education typically consume a healthier diet. However, with some aspects of the diet the opposite trend was seen: several papers reported that those with more education consume more sugar-sweetened beverages and more sodium. They also consume more multivitamin supplements. Income also seems to be positively associated with diet quality; however, only a handful of papers provided information on this. That persons with a higher SES consume a generally more nutritious diet is consistent with previous studies (2). Likewise, previous studies have also consistently reported that users of multivitamin supplements are generally better educated ^25– 27^.

The findings reported here indicate that failure to include SES in multivariate analysis of cohort studies may be a source of error due to residual confounding. There are several ways by which this might happen. For example, in studies of CVD, error may occur if people of higher SES have better access to the health-care system and are therefore more likely to be screened for risk factors for CVD and to then receive better quality preventive treatment (such as diagnosis and treatment of hypertension). Similarly, error may arise in studies where body weight is the end-point if those of higher SES make greater efforts than those of lower SES to avoid excess weight gain. Conversely, lower SES may increase the risk of disease via psychological pathways, such as by raising the level of stress or by inducing feelings of disempowerment.

In some cohort studies subjects are fairly similar with respect to SES. For example, the Physicians' Health Study included only male physicians ^28^. In such cases failure to adjust for SES is unlikely to lead to a significant error. However, in the large majority of cohort studies the subjects have a fairly wide variation in level of education and income.

Education, income, and social class (or occupation) has each been used as a covariate in the analysis of results from cohort studies. Education has been used far more often than the other two indicators of SES. While all three are closely associated with SES, they are quite distinct. For example, many people have a relatively poor education but still achieve a high income. This often occurs by marriage or by becoming a successful businessperson. Conversely, a person may have a college education but end up with a low income. It follows, therefore, that income (which indicates SES at the present time) may exert more influence on health and behavior than does education (which may have ceased 20 years or more before the cohort study started). Support for this possibility came from an Australian analysis that concluded that income was more strongly associated with diet than was education ^29^. These findings indicate, therefore, that as only a small minority of cohort studies (14.4% of the publications) included income as a covariate, this may be a significant source of error due to residual confounding. It is also possible that the reverse may be true. Education is stable throughout the lifespan and provides individuals with certain abilities related to learning and thinking which are important in matters of health. Measuring income of subjects recruited to a cohort study may often be problematic as many people refuse to divulge their income. For that reason occupation may be a more appropriate measure.

A related question is SES in childhood as this could play a significant role in disease risk in middle age or later. This was suggested by findings from a British cohort study ^3, 4^. Adjusting for a range of factors that indicate SES, both those in childhood and adulthood, attenuated the relationship between the plasma concentration of both vitamins C and E and risk of CHD in adults aged over 60. With the exception of that study, I am unaware of any other cohort study that has examined the relationship between SES in childhood and disease risk in adults older than age 50.

As cohort studies vary widely in such features as the country where they are conducted, the age and gender of subjects, the dietary variables being studied, and the health outcomes being investigated, it is likely that the magnitude of the error caused by residual confounding will also be highly variable. More research effort is required to determine the extent to which failure to adjust for SES across the lifecycle (as well as of other covariates) is a source of error in cohort studies.

Another potentially important covariate is growth in the fetal period and early infancy. Fetal development, as indicated by birthweight, as well as the rate of weight gain in early childhood, are predictors of risk of developing heart disease and type 2 diabetes 40 or 50 years later ^30^. While these variables may be only weakly related to SES, they are relevant here as they again underscore how diet, lifestyle, and SES in childhood (or even before birth) may be associated with disease risk decades later.

In conclusion, many cohort studies may have residual confounding caused by the failure to adjust for key covariates. Little attention seems to have been paid to this possible source of significant error. Of particular concern is the question of SES: roughly one third of papers have not included any measure of SES among the covariates. Education was used as the measure of SES far more often than income. However, education is likely to be a weaker measure of *present* SES than is income. There is a need for more research on the extent to which failure to include income (or another measure of present SES, such as occupation) leads to error in cohort studies.

## Supplementary material

**Papers used as a source of information for this study.**

Papers with the same number but different letters (e.g., 2a and 2b) indicate that the same cohort study contributed two separate papers
