# Supplementary material for: The possible importance of income and education as covariates in cohort studies that investigate the relationship between diet and disease
Source: F1000Res. 2016 May 18;4:690. Originally published 2015 Sep 7. [Version 2] doi: 10.12688/f1000research.6929.2 (PMC4897756; doi:10.12688/f1000research.6929.2)
Supplement: Supplementary file 1 [file f1000research-4-9431-s0000.tgz › 58c58252-7ff3-4cac-8841-1ddc7b241e71.docx]

**The possible importance of income and education as covariates in cohort studies**

**Supplementary material**

# Norman J Temple, PhD

The following is a list of all papers used as a source of information for the study. Papers with the same number but different letters (e.g., 2a and 2b) indicate that the same cohort study contributed two separate papers.

1. Voutilainen S, Rissanen TH, Virtanen J et al. [Low dietary folate intake is associated with an excess incidence of acute coronary events: The Kuopio Ischemic Heart Disease Risk Factor Study.](http://www.ncbi.nlm.nih.gov/pubmed/11390336) Circulation. 2001;103:2674-2680.

2a. [Drogan D](http://www.ncbi.nlm.nih.gov/pubmed/?term=Drogan%20D%5BAuthor%5D&cauthor=true&cauthor_uid=16870018), [Klipstein-Grobusch K](http://www.ncbi.nlm.nih.gov/pubmed/?term=Klipstein-Grobusch%20K%5BAuthor%5D&cauthor=true&cauthor_uid=16870018), [Dierkes J](http://www.ncbi.nlm.nih.gov/pubmed/?term=Dierkes%20J%5BAuthor%5D&cauthor=true&cauthor_uid=16870018) et al. Dietary intake of folate equivalents and risk of myocardial infarction in the European Prospective Investigation into Cancer and Nutrition (EPIC)--Potsdam study. [Public Health Nutr.](http://www.ncbi.nlm.nih.gov/pubmed?term=9%5Bvolume%5D+AND+465%5Bpage%5D+AND+drogan%5Bauthor%5D&cmd=detailssearch) 2006;9:465-471.

2b. Schulz M, Kroke A, Liese AD et al. [Food groups as predictors for short-term weight changes in men and women of the EPIC-Potsdam cohort.](http://www.ncbi.nlm.nih.gov/pubmed/12042455) J Nutr. 2002;132:1335-1340.

1. [Kurotani K](http://www.ncbi.nlm.nih.gov/pubmed/?term=Kurotani%20K%5BAuthor%5D&cauthor=true&cauthor_uid=23651531), [Nanri A](http://www.ncbi.nlm.nih.gov/pubmed/?term=Nanri%20A%5BAuthor%5D&cauthor=true&cauthor_uid=23651531), [Goto A](http://www.ncbi.nlm.nih.gov/pubmed/?term=Goto%20A%5BAuthor%5D&cauthor=true&cauthor_uid=23651531) et al. Red meat consumption is associated with the risk of type 2 diabetes in men but not in women: a Japan Public Health Center-based Prospective Study. [Br J Nutr.](http://www.ncbi.nlm.nih.gov/pubmed?term=110%5Bvolume%5D+AND+1910%5Bpage%5D&cmd=detailssearch) 2013;110:1910-1918.
2. Dalmeijer GW, Olthof MR, Verhoef P et al. [Prospective study on dietary intakes of folate, betaine, and choline and cardiovascular disease risk in women.](http://www.ncbi.nlm.nih.gov/pubmed/17375117) Eur J Clin Nutr. 2008;62:386-394.
3. Oomen CM, Feskens EJ, Räsänen L et al. [Fish consumption and coronary heart disease mortality in Finland, Italy, and The Netherlands.](http://www.ncbi.nlm.nih.gov/pubmed/10853639) Am J Epidemiol. 2000;151:999-1006.

6a. Pan A, Sun Q, Bernstein AM et al. [Changes in red meat consumption and subsequent risk of type 2 diabetes mellitus: three cohorts of US men and women.](http://www.ncbi.nlm.nih.gov/pubmed/23779232) JAMA Intern Med. 2013;173:1328-1335.

6b. Bao Y, Han J, Hu FB et al. [Association of nut consumption with total and cause-specific mortality.](http://www.ncbi.nlm.nih.gov/pubmed/24256379) N Engl J Med. 2013;369:2001-2011.

6c. Samieri C, Sun Q, Townsend MK et al. [The association between dietary patterns at midlife and health in aging: an observational study.](http://www.ncbi.nlm.nih.gov/pubmed/24189593) Ann Intern Med. 2013;159:584-589.

1. Mozaffarian D, Lemaitre RN, Kuller LH et al. [Cardiac benefits of fish consumption may depend on the type of fish meal consumed: the Cardiovascular Health Study.](http://www.ncbi.nlm.nih.gov/pubmed/12642356) Circulation. 2003;107:1372-1377.
2. Järvinen R, Knekt P, Rissanen H et al. [Intake of fish and long-chain n-3 fatty acids and the risk of coronary heart mortality in men and women.](http://www.ncbi.nlm.nih.gov/pubmed/16571163) Br J Nutr. 2006;95:824-829.

9a. Yamagishi K, Iso H, Date C et al. [Fish, omega-3 polyunsaturated fatty acids, and mortality from cardiovascular diseases in a nationwide community-based cohort of Japanese men and women the JACC (Japan Collaborative Cohort Study for Evaluation of Cancer Risk) Study.](http://www.ncbi.nlm.nih.gov/pubmed/18786479) J Am Coll Cardiol. 2008;52:988-996.

9b. Umesawa M, Iso H, Date C et al. [Relations between dietary sodium and potassium intakes and mortality from cardiovascular disease: the Japan Collaborative Cohort Study for Evaluation of Cancer Risks.](http://www.ncbi.nlm.nih.gov/pubmed/18614741) Am J Clin Nutr. 2008;88:195-202.

1. de Goede J, Geleijnse JM, Boer JM et al. [Marine (n-3) fatty acids, fish consumption, and the 10-year risk of fatal and nonfatal coronary heart disease in a large population of Dutch adults with low fish intake.](http://www.ncbi.nlm.nih.gov/pubmed/20335635) J Nutr. 2010;140:1023-1028.
2. Hendriksen MA, Boer JM, Du H et al. [No consistent association between consumption of energy-dense snack foods and annual weight and waist circumference changes in Dutch adults.](http://www.ncbi.nlm.nih.gov/pubmed/21613561) Am J Clin Nutr. 2011;94:19-25.
3. Tomasallo C, Anderson H, Haughwout M et al. [Mortality among frequent consumers of Great Lakes sport fish.](http://www.ncbi.nlm.nih.gov/pubmed/19811780) Environ Res. 2010;110:62-69.
4. Ludwig DS, Peterson KE, Gortmaker SL. [Relation between consumption of sugar-sweetened drinks and childhood obesity: a prospective, observational analysis.](http://www.ncbi.nlm.nih.gov/pubmed/11229668) Lancet. 2001;357:505-508.
5. Newby PK, Peterson KE, Berkey CS et al. [Beverage consumption is not associated with changes in weight and body mass index among low-income preschool children in North Dakota.](http://www.ncbi.nlm.nih.gov/pubmed/15215766) J Am Diet Assoc. 2004;104:1086-1094.
6. [Blum JW](http://www.ncbi.nlm.nih.gov/pubmed/?term=Blum%20JW%5BAuthor%5D&cauthor=true&cauthor_uid=15798075), [Jacobsen DJ](http://www.ncbi.nlm.nih.gov/pubmed/?term=Jacobsen%20DJ%5BAuthor%5D&cauthor=true&cauthor_uid=15798075), [Donnelly JE](http://www.ncbi.nlm.nih.gov/pubmed/?term=Donnelly%20JE%5BAuthor%5D&cauthor=true&cauthor_uid=15798075). Beverage consumption patterns in elementary school aged children across a two-year period. [J Am Coll Nutr.](http://www.ncbi.nlm.nih.gov/pubmed?term=24%5Bvolume%5D+AND+93%5Bpage%5D+AND+blum%5Bauthor%5D&cmd=detailssearch) 2005;24:93-98.

# [Mundt CA](http://www.ncbi.nlm.nih.gov/pubmed/?term=Mundt%20CA%5BAuthor%5D&cauthor=true&cauthor_uid=16826021), [Baxter-Jones AD](http://www.ncbi.nlm.nih.gov/pubmed/?term=Baxter-Jones%20AD%5BAuthor%5D&cauthor=true&cauthor_uid=16826021), [Whiting SJ](http://www.ncbi.nlm.nih.gov/pubmed/?term=Whiting%20SJ%5BAuthor%5D&cauthor=true&cauthor_uid=16826021) et al. Relationships of activity and sugar drink intake on fat mass development in youths. [Med Sci Sports Exerc.](http://www.ncbi.nlm.nih.gov/pubmed?term=1245%5Bpage%5D+AND+mundt%5Bauthor%5D&cmd=detailssearch) 2006;38:1245-1254.

1. Striegel-Moore RH, Thompson D, Affenito SG et al. [Correlates of beverage intake in adolescent girls: the National Heart, Lung, and Blood Institute Growth and Health Study.](http://www.ncbi.nlm.nih.gov/pubmed/16492426) J Pediatr. 2006;148:183-187.
2. [Johnson L](http://www.ncbi.nlm.nih.gov/pubmed/?term=Johnson%20L%5BAuthor%5D&cauthor=true&cauthor_uid=17616342), [Mander AP](http://www.ncbi.nlm.nih.gov/pubmed/?term=Mander%20AP%5BAuthor%5D&cauthor=true&cauthor_uid=17616342), [Jones LR](http://www.ncbi.nlm.nih.gov/pubmed/?term=Jones%20LR%5BAuthor%5D&cauthor=true&cauthor_uid=17616342) et al. Is sugar-sweetened beverage consumption associated with increased fatness in children? [Nutrition.](http://www.ncbi.nlm.nih.gov/pubmed?term=23%5Bvolume%5D+AND+557%5Bpage%5D+AND+johnson%5Bauthor%5D&cmd=detailssearch) 2007;23:557-563.
3. Laurson K, Eisenmann JC, Moore S. [Lack of association between television viewing, soft drinks, physical activity and body mass index in children.](http://www.ncbi.nlm.nih.gov/pubmed/18410467) Acta Paediatr. 2008;97:795-800.
4. Libuda L, Alexy U, Sichert-Hellert W et al. [Pattern of beverage consumption and long-term association with body-weight status in German adolescents--results from the DONALD study.](http://www.ncbi.nlm.nih.gov/pubmed/18034911) Br J Nutr. 2008;99:1370-1379.
5. Vanselow MS, Pereira MA, Neumark-Sztainer D et al. [Adolescent beverage habits and changes in weight over time: findings from Project EAT.](http://www.ncbi.nlm.nih.gov/pubmed/19864412) Am J Clin Nutr. 2009;90:1489-1495.
6. [Carlson JA](http://www.ncbi.nlm.nih.gov/pubmed/?term=Carlson%20JA%5BAuthor%5D&cauthor=true&cauthor_uid=22799510), [Crespo NC](http://www.ncbi.nlm.nih.gov/pubmed/?term=Crespo%20NC%5BAuthor%5D&cauthor=true&cauthor_uid=22799510), [Sallis JF](http://www.ncbi.nlm.nih.gov/pubmed/?term=Sallis%20JF%5BAuthor%5D&cauthor=true&cauthor_uid=22799510) et al. Dietary-related and physical activity-related predictors of obesity in children: a 2-year prospective study. [Child Obes.](http://www.ncbi.nlm.nih.gov/pubmed?term=8%5Bvolume%5D+AND+110%5Bpage%5D+AND+carlson%5Bauthor%5D&cmd=detailssearch) 2012;8:110-115.
7. [Laska MN](http://www.ncbi.nlm.nih.gov/pubmed/?term=Laska%20MN%5BAuthor%5D&cauthor=true&cauthor_uid=21701567), [Murray DM](http://www.ncbi.nlm.nih.gov/pubmed/?term=Murray%20DM%5BAuthor%5D&cauthor=true&cauthor_uid=21701567), [Lytle LA](http://www.ncbi.nlm.nih.gov/pubmed/?term=Lytle%20LA%5BAuthor%5D&cauthor=true&cauthor_uid=21701567) et al. Longitudinal associations between key dietary behaviors and weight gain over time: transitions through the adolescent years. [Obesity (Silver Spring).](http://www.ncbi.nlm.nih.gov/pubmed?term=20%5Bvolume%5D+AND+118%5Bpage%5D+AND+laska%5Bauthor%5D&cmd=detailssearch) 2012;20:118-125.
8. [Olsen NJ](http://www.ncbi.nlm.nih.gov/pubmed/?term=Olsen%20NJ%5BAuthor%5D&cauthor=true&cauthor_uid=22854439), [Andersen LB](http://www.ncbi.nlm.nih.gov/pubmed/?term=Andersen%20LB%5BAuthor%5D&cauthor=true&cauthor_uid=22854439), [Wedderkopp N](http://www.ncbi.nlm.nih.gov/pubmed/?term=Wedderkopp%20N%5BAuthor%5D&cauthor=true&cauthor_uid=22854439) et al. Intake of liquid and solid sucrose in relation to changes in body fatness over 6 years among 8- to 10-year-old children: the European Youth Heart Study. [Obes Facts.](http://www.ncbi.nlm.nih.gov/pubmed?term=5%5Bvolume%5D+AND+506%5Bpage%5D+AND+olsen%5Bauthor%5D&cmd=detailssearch) 2012;5:506-512.

25a. Palmer JR, Boggs DA, Krishnan S et al. [Sugar-sweetened beverages and incidence of type 2 diabetes mellitus in African American women.](http://www.ncbi.nlm.nih.gov/pubmed/18663160) Arch Intern Med. 2008;168:1487-1492.

25b. [Boggs DA](http://www.ncbi.nlm.nih.gov/pubmed/?term=Boggs%20DA%5BAuthor%5D&cauthor=true&cauthor_uid=23902954), [Rosenberg L](http://www.ncbi.nlm.nih.gov/pubmed/?term=Rosenberg%20L%5BAuthor%5D&cauthor=true&cauthor_uid=23902954), [Rodríguez-Bernal CL](http://www.ncbi.nlm.nih.gov/pubmed/?term=Rodr%C3%ADguez-Bernal%20CL%5BAuthor%5D&cauthor=true&cauthor_uid=23902954) et al. Long-term diet quality is associated with lower obesity risk in young African American women with normal BMI at baseline. [J Nutr.](http://www.ncbi.nlm.nih.gov/pubmed?term=143%5Bvolume%5D+AND+1636%5Bpage%5D+AND+boggs%5Bauthor%5D&cmd=detailssearch) 2013;143:1636-1641.

1. Stookey JD, Constant F, Popkin BM et al. [Drinking water is associated with weight loss in overweight dieting women independent of diet and activity.](http://www.ncbi.nlm.nih.gov/pubmed/18787524) Obesity (Silver Spring). 2008;16:2481-2488.
2. Bes-Rastrollo M, Sanchez-Villegas A, Basterra-Gortari FJ et al. [Prospective study of self-reported usual snacking and weight gain in a Mediterranean cohort: the SUN project.](http://www.ncbi.nlm.nih.gov/pubmed/19748710) Clin Nutr. 2010;29:323-330
3. Jacques PF, Cassidy A, Rogers G et al. [Higher dietary flavonol intake is associated with lower incidence of type 2 diabetes.](http://www.ncbi.nlm.nih.gov/pubmed/23902957) J Nutr. 2013;143:1474-1480.
4. [Drapeau V](http://www.ncbi.nlm.nih.gov/pubmed/?term=Drapeau%20V%5BAuthor%5D&cauthor=true&cauthor_uid=15213024), [Després JP](http://www.ncbi.nlm.nih.gov/pubmed/?term=Despr%C3%A9s%20JP%5BAuthor%5D&cauthor=true&cauthor_uid=15213024), [Bouchard C](http://www.ncbi.nlm.nih.gov/pubmed/?term=Bouchard%20C%5BAuthor%5D&cauthor=true&cauthor_uid=15213024) et al. Modifications in food-group consumption are related to long-term body-weight changes. [Am J Clin Nutr.](http://www.ncbi.nlm.nih.gov/pubmed?term=80%5Bvolume%5D+AND+29%5Bpage%5D+AND+drapeau%5Bauthor%5D&cmd=detailssearch) 2004;80:29-37.
5. [Halkjaer J](http://www.ncbi.nlm.nih.gov/pubmed/?term=Halkjaer%20J%5BAuthor%5D&cauthor=true&cauthor_uid=15522143), [Sørensen TI](http://www.ncbi.nlm.nih.gov/pubmed/?term=S%C3%B8rensen%20TI%5BAuthor%5D&cauthor=true&cauthor_uid=15522143), [Tjønneland A](http://www.ncbi.nlm.nih.gov/pubmed/?term=Tj%C3%B8nneland%20A%5BAuthor%5D&cauthor=true&cauthor_uid=15522143) et al. Food and drinking patterns as predictors of 6-year BMI-adjusted changes in waist circumference. [Br J Nutr.](http://www.ncbi.nlm.nih.gov/pubmed?term=92%5Bvolume%5D+AND+735%5Bpage%5D+AND+Halkjaer%5Bauthor%5D&cmd=detailssearch) 2004;92:735-748.
6. Halkjaer J, Tjønneland A, Overvad K et al. [Dietary predictors of 5-year changes in waist circumference.](http://www.ncbi.nlm.nih.gov/pubmed/19631041) J Am Diet Assoc. 2009;109:1356-1366.
7. [Kvaavik E](http://www.ncbi.nlm.nih.gov/pubmed/?term=Kvaavik%20E%5BAuthor%5D&cauthor=true&cauthor_uid=15877908), [Andersen LF](http://www.ncbi.nlm.nih.gov/pubmed/?term=Andersen%20LF%5BAuthor%5D&cauthor=true&cauthor_uid=15877908), [Klepp KI](http://www.ncbi.nlm.nih.gov/pubmed/?term=Klepp%20KI%5BAuthor%5D&cauthor=true&cauthor_uid=15877908). The stability of soft drinks intake from adolescence to adult age and the association between long-term consumption of soft drinks and lifestyle factors and body weight. [Public Health Nutr.](http://www.ncbi.nlm.nih.gov/pubmed?term=Public+health+nutrition%5BJour%5D+AND+8%5Bvolume%5D+AND+149%5Bpage%5D&cmd=detailssearch) 2005;8:149-157.
8. Odegaard AO, Koh WP, Arakawa K et al. [Soft drink and juice consumption and risk of physician-diagnosed incident type 2 diabetes: the Singapore Chinese Health Study.](http://www.ncbi.nlm.nih.gov/pubmed/20160170) Am J Epidemiol. 2010;171:701-708.
9. [Li K](http://www.ncbi.nlm.nih.gov/pubmed?term=Li%20K%5BAuthor%5D&cauthor=true&cauthor_uid=21779961), [Kaaks R](http://www.ncbi.nlm.nih.gov/pubmed?term=Kaaks%20R%5BAuthor%5D&cauthor=true&cauthor_uid=21779961), [Linseisen J](http://www.ncbi.nlm.nih.gov/pubmed?term=Linseisen%20J%5BAuthor%5D&cauthor=true&cauthor_uid=21779961) et al. Vitamin/mineral supplementation and cancer, cardiovascular, and all-cause mortality in a German prospective cohort (EPIC-Heidelberg). [Eur J Nutr.](http://www.ncbi.nlm.nih.gov/pubmed?term=51%5Bvolume%5D+AND+407%5Bpage%5D+AND+li%5Bauthor%5D&cmd=detailssearch) 2012;51:407-413.
10. Muntwyler J, Hennekens CH, Manson JE et al. [Vitamin supplement use in a low-risk population of US male physicians and subsequent cardiovascular mortality.](http://www.ncbi.nlm.nih.gov/pubmed/12090883) Arch Intern Med. 2002;162:1472-1476.

36a. Mursu J, Robien K, Harnack LJ et al. [Dietary supplements and mortality rate in older women: the Iowa Women's Health Study.](http://www.ncbi.nlm.nih.gov/pubmed/21987192) Arch Intern Med. 2011;171:1625-1633.

36b. Inoue-Choi M, Robien K, Mariani A et al. [Sugar-sweetened beverage intake and the risk of type I and type II endometrial cancer among postmenopausal women.](http://www.ncbi.nlm.nih.gov/pubmed/24273064) Cancer Epidemiol Biomarkers Prev. 2013;22:2384-2394.

36c. [Folsom AR](http://www.ncbi.nlm.nih.gov/pubmed?term=Folsom%20AR%5BAuthor%5D&cauthor=true&cauthor_uid=15522857), [Demissie Z](http://www.ncbi.nlm.nih.gov/pubmed?term=Demissie%20Z%5BAuthor%5D&cauthor=true&cauthor_uid=15522857). Fish intake, marine omega-3 fatty acids, and mortality in a cohort of postmenopausal women. [Am J Epidemiol.](http://www.ncbi.nlm.nih.gov/pubmed?term=160%5Bvolume%5D+AND+1005%5Bpage%5D+AND+Folsom%5Bauthor%5D&cmd=detailssearch) 2004;160:1005-1010.

1. [Neuhouser ML, Wassertheil-Smoller S, Thomson C et al.](http://www.ncbi.nlm.nih.gov/pubmed/19204221) Multivitamin use and risk of cancer and cardiovascular disease in the Women's Health Initiative cohorts. Arch Intern Med. 2009;169:294-304.

38a. [Park SY](http://www.ncbi.nlm.nih.gov/pubmed?term=Park%20SY%5BAuthor%5D&cauthor=true&cauthor_uid=21343248), [Murphy SP](http://www.ncbi.nlm.nih.gov/pubmed?term=Murphy%20SP%5BAuthor%5D&cauthor=true&cauthor_uid=21343248), [Wilkens LR](http://www.ncbi.nlm.nih.gov/pubmed?term=Wilkens%20LR%5BAuthor%5D&cauthor=true&cauthor_uid=21343248) et al. Multivitamin use and the risk of mortality and cancer incidence: the multiethnic cohort study. [Am J Epidemiol.](http://www.ncbi.nlm.nih.gov/pubmed?term=173%5Bvolume%5D+AND+906%5Bpage%5D+AND+park%5Bauthor%5D&cmd=detailssearch) 2011;173:906-914.

38b. Park SY, Ollberding NJ, Woolcott CG et al. [Fruit and vegetable intakes are associated with lower risk of bladder cancer among women in the Multiethnic Cohort Study.](http://www.ncbi.nlm.nih.gov/pubmed/23739308) J Nutr. 2013;143:1283-1292

39a. Pocobelli G, Peters U, Kristal AR et al. [Use of supplements of multivitamins, vitamin C, and vitamin E in relation to mortality.](http://www.ncbi.nlm.nih.gov/pubmed/19596711) Am J Epidemiol. 2009;170:472-483.

39b. Perrigue MM, Kantor ED, Hastert TA et al. [Eating frequency and risk of colorectal cancer.](http://www.ncbi.nlm.nih.gov/pubmed/24057417) Cancer Causes Control. 2013;24:2107-2115.

40a. Rautiainen S, Akesson A, Levitan EB et al. [Multivitamin use and the risk of myocardial infarction: a population-based cohort of Swedish women.](http://www.ncbi.nlm.nih.gov/pubmed/20861174) Am J Clin Nutr. 2010;92:1251-1256.

40b. Männistö S, Dixon LB, Balder HF et al. [Dietary patterns and breast cancer risk: results from three cohort studies in the DIETSCAN project.](http://www.ncbi.nlm.nih.gov/pubmed/16049811) Cancer Causes Control. 2005;16:725-733.

1. [Watkins ML](http://www.ncbi.nlm.nih.gov/pubmed?term=Watkins%20ML%5BAuthor%5D&cauthor=true&cauthor_uid=10909952), [Erickson JD](http://www.ncbi.nlm.nih.gov/pubmed?term=Erickson%20JD%5BAuthor%5D&cauthor=true&cauthor_uid=10909952), [Thun MJ](http://www.ncbi.nlm.nih.gov/pubmed?term=Thun%20MJ%5BAuthor%5D&cauthor=true&cauthor_uid=10909952) et al. Multivitamin use and mortality in a large prospective study. [Am J Epidemiol.](http://www.ncbi.nlm.nih.gov/pubmed?term=152%5Bvolume%5D+AND+149%5Bpage%5D+AND+Watkins%5Bauthor%5D&cmd=detailssearch) 2000;152:149-162.
2. Thomas LD, Elinder CG, Tiselius HG et al. [Ascorbic acid supplements and kidney stone incidence among men: a prospective study.](http://www.ncbi.nlm.nih.gov/pubmed/23381591) JAMA Intern Med. 2013;173:386-388.
3. Stoof SP, Twisk JW, Olthof MR. I[s the intake of sugar-containing beverages during adolescence related to adult weight status?](http://www.ncbi.nlm.nih.gov/pubmed/22067526) Public Health Nutr. 2013;16:1257-1262.
4. Barrio-Lopez MT, Martinez-Gonzalez MA, Fernandez-Montero A et al. [Prospective study of changes in sugar-sweetened beverage consumption and the incidence of the metabolic syndrome and its components: the SUN cohort.](http://www.ncbi.nlm.nih.gov/pubmed/23534417) Br J Nutr. 2013;110:1722-1731.
5. Wengreen H, Munger RG, Cutler A et al. [Prospective study of Dietary Approaches to Stop Hypertension- and Mediterranean-style dietary patterns and age-related cognitive change: the Cache County Study on Memory, Health and Aging.](http://www.ncbi.nlm.nih.gov/pubmed/24047922) Am J Clin Nutr. 2013;98:1263-1271.
6. Link LB, Canchola AJ, Bernstein L et al. [Dietary patterns and breast cancer risk in the California Teachers Study cohort.](http://www.ncbi.nlm.nih.gov/pubmed/24108781) Am J Clin Nutr. 2013;98:1524-1532.
7. Mood C. Life-style and self-rated global health in Sweden: a prospective analysis spanning three decades. [Prev Med.](http://www.ncbi.nlm.nih.gov/pubmed?term=57%5Bvolume%5D+AND+802%5Bpage%5D+AND+mood%5Bauthor%5D&cmd=detailssearch) 2013;57:802-806.
8. Dominianni C, Huang WY, Berndt S et al. [Prospective study of the relationship between coffee and tea with colorectal cancer risk: the PLCO Cancer Screening Trial.](http://www.ncbi.nlm.nih.gov/pubmed/23907431) Br J Cancer. 2013;109:1352-1359.
9. [Gardener H](http://www.ncbi.nlm.nih.gov/pubmed/?term=Gardener%20H%5BAuthor%5D&cauthor=true&cauthor_uid=23784068), [Rundek T](http://www.ncbi.nlm.nih.gov/pubmed/?term=Rundek%20T%5BAuthor%5D&cauthor=true&cauthor_uid=23784068), [Wright CB](http://www.ncbi.nlm.nih.gov/pubmed/?term=Wright%20CB%5BAuthor%5D&cauthor=true&cauthor_uid=23784068) et al. Coffee and tea consumption are inversely associated with mortality in a multiethnic urban population. [J Nutr.](http://www.ncbi.nlm.nih.gov/pubmed?term=1299%5Bpage%5D+AND+Gardener%5Bauthor%5D&cmd=detailssearch) 2013;143:1299-1308.

50a. Xiao Q, Murphy RA, Houston DK et al. [Dietary and supplemental calcium intake and cardiovascular disease mortality: the National Institutes of Health-AARP diet and health study.](http://www.ncbi.nlm.nih.gov/pubmed/23381719) JAMA Intern Med. 2013;173:639-646

50b. Sinha R, Cross AJ, Graubard BI et al. [Meat intake and mortality: a prospective study of over half a million people.](http://www.ncbi.nlm.nih.gov/pubmed/19307518) Arch Intern Med. 2009;169:562-571.

1. [Oomen CM](http://www.ncbi.nlm.nih.gov/pubmed/?term=Oomen%20CM%5BAuthor%5D&cauthor=true&cauthor_uid=11253967), [Ocké MC](http://www.ncbi.nlm.nih.gov/pubmed/?term=Ock%C3%A9%20MC%5BAuthor%5D&cauthor=true&cauthor_uid=11253967), [Feskens EJ](http://www.ncbi.nlm.nih.gov/pubmed/?term=Feskens%20EJ%5BAuthor%5D&cauthor=true&cauthor_uid=11253967) et al. Association between trans fatty acid intake and 10-year risk of coronary heart disease in the Zutphen Elderly Study: a prospective population-based study. [Lancet.](http://www.ncbi.nlm.nih.gov/pubmed?term=357%5Bvolume%5D+AND+746%5Bpage%5D&cmd=detailssearch) 2001;357:746-751.

52a. [Rohrmann S](http://www.ncbi.nlm.nih.gov/pubmed?term=Rohrmann%20S%5BAuthor%5D&cauthor=true&cauthor_uid=23497300), [Overvad K](http://www.ncbi.nlm.nih.gov/pubmed?term=Overvad%20K%5BAuthor%5D&cauthor=true&cauthor_uid=23497300), [Bueno-de-Mesquita HB](http://www.ncbi.nlm.nih.gov/pubmed?term=Bueno-de-Mesquita%20HB%5BAuthor%5D&cauthor=true&cauthor_uid=23497300) et al. Meat consumption and mortality--results from the European Prospective Investigation into Cancer and Nutrition. [BMC Med.](http://www.ncbi.nlm.nih.gov/pubmed?term=11%5Bvolume%5D+AND+63%5Bpage%5D+AND+Rohrmann%5Bauthor%5D&cmd=detailssearch) 2013;11:63.

52b. Leenders M, Sluijs I, Ros MM et al. [Fruit and vegetable consumption and mortality: European prospective investigation into cancer and nutrition.](http://www.ncbi.nlm.nih.gov/pubmed/23599238) Am J Epidemiol. 2013;178:590-602.

1. Golley RK, Smithers LG, Mittinty MN et al. [Diet quality of U.K. infants is associated with dietary, adiposity, cardiovascular, and cognitive outcomes measured at 7-8 years of age.](http://www.ncbi.nlm.nih.gov/pubmed/23946339) J Nutr. 2013;143:1611-1617.
2. Parrott MD, Shatenstein B, Ferland G et al. [Relationship between diet quality and cognition depends on socioeconomic position in healthy older adults.](http://www.ncbi.nlm.nih.gov/pubmed/23986363) J Nutr. 2013;143:1767-1773.

55a. [Kweon SS](http://www.ncbi.nlm.nih.gov/pubmed/?term=Kweon%20SS%5BAuthor%5D&cauthor=true&cauthor_uid=23986366), [Shu XO](http://www.ncbi.nlm.nih.gov/pubmed/?term=Shu%20XO%5BAuthor%5D&cauthor=true&cauthor_uid=23986366), [Xiang Y](http://www.ncbi.nlm.nih.gov/pubmed/?term=Xiang%20Y%5BAuthor%5D&cauthor=true&cauthor_uid=23986366) et al. Intake of specific nonfermented soy foods may be inversely associated with risk of distal gastric cancer in a Chinese population. [J Nutr.](http://www.ncbi.nlm.nih.gov/pubmed?term=143%5Bvolume%5D+AND+1736%5Bpage%5D&cmd=detailssearch) 2013;143:1736-1742.

55b. Yu D, Shu XO, Li H et al. [Dietary carbohydrates, refined grains, glycemic load, and risk of coronary heart disease in Chinese adults.](http://www.ncbi.nlm.nih.gov/pubmed/24008907) Am J Epidemiol. 2013;178:1542-1549.

1. [Velie EM](http://www.ncbi.nlm.nih.gov/pubmed?term=Velie%20EM%5BAuthor%5D&cauthor=true&cauthor_uid=16332665), [Schairer C](http://www.ncbi.nlm.nih.gov/pubmed?term=Schairer%20C%5BAuthor%5D&cauthor=true&cauthor_uid=16332665), [Flood A](http://www.ncbi.nlm.nih.gov/pubmed?term=Flood%20A%5BAuthor%5D&cauthor=true&cauthor_uid=16332665) et al. Empirically derived dietary patterns and risk of postmenopausal breast cancer in a large prospective cohort study. [Am J Clin Nutr.](http://www.ncbi.nlm.nih.gov/pubmed?term=82%5Bvolume%5D+AND+1308%5Bpage%5D+AND+velie%5Bauthor%5D&cmd=detailssearch) 2005;82:1308-1319.
2. Cottet V, Touvier M, Fournier A et al. [Postmenopausal breast cancer risk and dietary patterns in the E3N-EPIC prospective cohort study.](http://www.ncbi.nlm.nih.gov/pubmed/19828509) Am J Epidemiol. 2009;170:1257-1267.
3. [Tuomilehto J](http://www.ncbi.nlm.nih.gov/pubmed/?term=Tuomilehto%20J%5BAuthor%5D&cauthor=true&cauthor_uid=11265954), [Jousilahti P](http://www.ncbi.nlm.nih.gov/pubmed/?term=Jousilahti%20P%5BAuthor%5D&cauthor=true&cauthor_uid=11265954), [Rastenyte D](http://www.ncbi.nlm.nih.gov/pubmed/?term=Rastenyte%20D%5BAuthor%5D&cauthor=true&cauthor_uid=11265954) et al. Urinary sodium excretion and cardiovascular mortality in Finland: a prospective study. [Lancet.](http://www.ncbi.nlm.nih.gov/pubmed?term=357%5Bvolume%5D+AND+848%5Bpage%5D&cmd=detailssearch) 2001;357:848-851.
4. Nagata C, Takatsuka N, Shimizu N et al. [Sodium intake and risk of death from stroke in Japanese men and women.](http://www.ncbi.nlm.nih.gov/pubmed/15143292) Stroke. 2004;35:1543-1547.
5. Cohen HW, Hailpern SM, Fang J et al. [Sodium intake and mortality in the NHANES II follow-up study.](http://www.ncbi.nlm.nih.gov/pubmed/16490476) Am J Med. 2006;119:275.e7-14.
6. [Cohen HW](http://www.ncbi.nlm.nih.gov/pubmed?term=Cohen%20HW%5BAuthor%5D&cauthor=true&cauthor_uid=18465175), [Hailpern SM](http://www.ncbi.nlm.nih.gov/pubmed?term=Hailpern%20SM%5BAuthor%5D&cauthor=true&cauthor_uid=18465175), [Alderman MH](http://www.ncbi.nlm.nih.gov/pubmed?term=Alderman%20MH%5BAuthor%5D&cauthor=true&cauthor_uid=18465175). Sodium intake and mortality follow-up in the Third National Health and Nutrition Examination Survey (NHANES III). [J Gen Intern Med.](http://www.ncbi.nlm.nih.gov/pubmed?term=23%5Bvolume%5D+AND+1297%5Bpage%5D+AND+cohen%5Bauthor%5D&cmd=detailssearch) 2008;23:1297-1302.
7. Larsson SC, Virtanen MJ, Mars M et al. [Magnesium, calcium, potassium, and sodium intakes and risk of stroke in male smokers.](http://www.ncbi.nlm.nih.gov/pubmed/18332289) Arch Intern Med. 2008;168:459-465.
