# Supplementary material for: The possible importance of income and education as covariates in cohort studies that investigate the relationship between diet and disease
Source: F1000Res. 2016 May 18;4:690. Originally published 2015 Sep 7. [Version 2] doi: 10.12688/f1000research.6929.2 (PMC4897756; doi:10.12688/f1000research.6929.2)
Supplement: Peer review history

# Peer review: Referee response for version 2

**DOI:** 10.5256/f1000research.9431.r14208

**Published:** 2016-06-06

**Authors:** Tord Finne Vedøy

**Affiliations:** 1 Norwegian Institute for Alcohol and Drug Research, Oslo, Norway

I have re-read the paper and I find that the author has addressed the issues I raised in my first review. I therefore approve this paper.

I have read this submission. I believe that I have an appropriate level of expertise to confirm that it is of an acceptable scientific standard.

# Peer review: Referee response for version 1

**DOI:** 10.5256/f1000research.7462.r13500

**Published:** 2016-05-12

**Authors:** Silvano Gallus

**Affiliations:** 1 Department of Epidemiology, IRCCS-Mario Negri Institute for Pharmacological Research, Milan, Italy

This is a well-written manuscript providing information on the use of socioeconomic status (SES) measures as covariates in 76 papers from 66 cohort studies. The study is interesting and original. I only have a few minor points which could improve the presentation of findings:

1. The objectives of the study may be clarified in the background or methods sections of the abstract;
2. In the conclusions of the abstract, I suggest to delete the term "therefore" (2 ^nd^ line of the Conclusions);
3. In population-based cohort studies, income may represent a major problem, given the likely high nonresponse rate. For example, in a European cross-sectional study I conducted in 2010 on more than 18,000 European adults, 22% of participants did not provide information on income, and only a negligible proportion of subjects did not provide information on education. Also for this reason, education (or alternatively occupation) may be considered a more appropriate proxy of SES than income. This issue may be further addressed both in the abstract and in the main text.

I have read this submission. I believe that I have an appropriate level of expertise to confirm that it is of an acceptable scientific standard.

# Peer review: Referee response for version 1

**DOI:** 10.5256/f1000research.7462.r11155

**Published:** 2015-11-18

**Authors:** Tord Finne Vedøy

**Affiliations:** 1 Norwegian Institute for Alcohol and Drug Research, Oslo, Norway

This paper is an important reminder that SES is a crucial factor to consider when doing research on health related lifestyle issues. However, I do have some comments of minor importance.

First, in the first paragraph of the introduction the author emphasizes the role of a nutritious diet for a health lifestyle. Although I understand that the paper focuses on nutrition, it could be noted that smoking still is one of the largest, if not the largest, single cause of death and disease in most developed countries. For the same reasons it might be a good idea to mention in the title that the focus of the paper is nutrition.

Second, the author should make explicit what is meant by “cohort studies”. Does this refer to panel-studies, age-period-cohort studies or both?

Third, the transition from the first to the second paragraph in the introduction seems wanting. As I read it, it is the pattern (“that people with a higher SES consume a more nutritious diet”) that “suggests that income, or other indicators of SES, may be a relevant factor in cohort studies”. However, if cohort is to be an important factor, the temporal or historical dimension of the pattern should be emphasized.

Fourth, the discussion of the relative importance of different measures of SES on health could be more nuanced. While there may be good reasons for using income rather than education as a measure of SES, especially in matters of consumption, education has certain characteristics which income lacks. For example, education is stable throughout the lifespan and provides individuals with certain abilities related to learning and thinking which are important in matters of health. These ideas has been discussed by for example Cutler and Lleras-Muney (2010).

I have read this submission. I believe that I have an appropriate level of expertise to confirm that it is of an acceptable scientific standard, however I have significant reservations, as outlined above.

## References

[1] CutlerDMLleras-MuneyA: Understanding differences in health behaviors by education. *J Health Econ*.2010;29(1) : 10.1016/j.jhealeco.2009.10.003 1-28 10.1016/j.jhealeco.2009.10.003 19963292PMC2824018

# Response

**Published:** 2016-05-08

**Authors:** Norman Temple

**Affiliations:** Athabasca University, Canada

I thank Dr Vedøy for his helpful comments. I have made edits to the paper based on those comments.
